# Chemical Profiles and Toxicity of Electronic Cigarettes: An Umbrella Review and Methodological Considerations

**DOI:** 10.3390/ijerph20031908

**Published:** 2023-01-20

**Authors:** Nargiz Travis, Marie Knoll, Steven Cook, Hayoung Oh, Christopher J. Cadham, Luz María Sánchez-Romero, David T. Levy

**Affiliations:** 1 Lombardi Comprehensive Cancer Center, Georgetown Medical University, Washington, DC 20007, USA; 2 Department of Epidemiology, School of Public Health, University of Michigan, Ann Arbor, MI 48104, USA; 3 Department of Health Management and Policy, School of Public Health, University of Michigan, Ann Arbor, MI 48104, USA

**Keywords:** umbrella review, toxicity, chemical profiles, constituents, electronic cigarettes, vaping

## Abstract

Background: Electronic cigarettes (ECs) are often marketed as a safer alternative to combustible tobacco products. The global EC market has rapidly expanded since their introduction, creating an urgent need for research describing the toxicity and chemical composition of ECs. We conducted an umbrella review to summarize the evidence from existing systematic reviews (SRs). Methods: The search for SRs was conducted across four electronic databases through 25 January 2022. Methodological quality was assessed using the AMSTAR-2 quality appraisal tool. Results: Twenty-five SRs were included in our umbrella review. Chemical profiles widely varied across studies included in the reviews, which was mainly attributed to the lack of standardized protocols investigating the constituents, and differences in EC devices and e-liquids tested. Metals were more abundant in some EC aerosols than cigarettes, while carbonyls were typically found at lower levels. There was consistent evidence of in vitro toxicity from EC aerosol and e-liquid exposure. AMSTAR-2 revealed important limitations across reviews. Conclusions: While most reviews concluded that ECs were likely less harmful than cigarettes, there was hesitancy to draw clear conclusions due to variable analytical procedures and inconsistent findings among the included studies. Future SRs with improved methodology and reporting are needed to adequately inform tobacco regulatory actions.

## 1. Introduction

The electronic cigarette (EC) is a nicotine delivery system that is often marketed as a safer alternative to combustible tobacco products [1]. ECs have been constantly evolving since their invention in 2003, with mainly four types of devices available to the user: disposable cig-a-likes prefilled with a liquid (i.e., first-generation devices), prefilled or refillable cartridges (i.e., second-generation devices), refillable and modifiable tank cartridges, also known as “mods”, that allow for a change in voltage or power, (i.e., third-generation devices), and prefilled or refillable pod cartridges with a modifiable system, also known as “pod-mods” (i.e., fourth-generation devices) [2].

Unlike conventional cigarettes (CC), which involve tobacco combustion, exposing users to thousands of chemicals, ECs heat and aerosolize liquid generally containing propylene glycol, vegetable glycerin, nicotine (in nicotine-containing solutions), flavorings (in flavored solutions), and other chemicals [3,4]. The thermal degradation of the EC liquid (e-liquid) during the heating process emits potentially harmful compounds, such as heavy metals, low molecular weight carbonyl compounds (e.g., formaldehyde, acetaldehyde, and acetone), and tobacco-specific nitrosamines [5]. However, with aerosol constituents and their emission levels varying across different EC devices, mixed information exists regarding their likely chemistry [6].

With the rapid expansion of the global EC market, reliable information is needed regarding the chemical composition and the potentially toxic effects of ECs so that evidence-based regulations can limit the harm associated with EC use. Given limited short-term and lack of long-term data on human health outcomes [7], an important source of EC risk assessment relies on the results from toxicity studies [8,9]. Toxicity assessments are commonplace in tobacco research, since they can help identify levels of exposure that are safe for humans [8]. Such assessments are also essential for testing and understanding the mechanisms by which chemicals affect animals and humans at the cellular level, thereby bridging the gap between in vitro and in vivo studies.

An emergent body of research has examined the chemical profiles (i.e., chemical elements contained in EC aerosol and/or e-liquid) and toxicity (i.e., in vitro testing of cytotoxic and genotoxic effects on cultured cells exposed to aerosol or e-liquid) of ECs, and several systematic reviews have attempted to synthesize the findings from these studies. However, these reviews produced heterogeneous results, making it important to summarize the findings and areas of disagreement of existing systematic reviews and critically evaluate their methodological quality. In this umbrella review, we aim to summarize the main findings, conclusions, and recommendations of these reviews, critically assess their methodological quality, highlight limitations of current research, and identify needs for future research.

## 2. Methods

Our review protocol was developed a priori in adherence with the guidelines for umbrella reviews [10], and registered with PROSPERO (Registration Number: CRD42021237878). The original review protocol also planned to include evidence on health outcomes of ECs. However, the volume of the literature and the significance of differences related to the chemical constituents and EC toxicity literature warranted two separate publications. The present paper is an extension of our previously published umbrella review on the health effects of ECs and follows the same methodology [7]. It covers chemical profiles and toxicity of ECs and was reported in adherence with the Preferred Reporting Items for Systematic reviews and Meta-analyses (PRISMA) [11].

### 2.1. Search Strategy and Terms

A structured literature search was conducted on 27 May 2020 in PubMed, Web of Science, Embase, and Cochrane Database of Systematic Reviews by two reviewers (NT, CJC) and was updated on 25 January 2022. The search terms included “electronic cigarette”, “e-cigarette”, “electronic nicotine delivery system”, “personal vaporiser”, “personal vaporizer”, “e-liquid”, “nicotine content”, “systematic review”, “meta-analysis”, and “review”. The complete search strategy has been previously described in more detail in our earlier umbrella review [7], and the PubMed database search is available in Supplementary Table S1.

### 2.2. Eligibility

This umbrella review included systematic reviews and meta-analyses published in English that examined outcomes related to: (1) chemical profiles of e-liquids/EC aerosols, or (2) toxicity of e-liquids/EC aerosols. We considered a literature review to be systematic if it explicitly identified itself as such in the title, abstract, keyword, or methods. Editorials, commentaries, and letters to the editor were excluded. Evidence from experimental chemical and in vitro human and animal cell studies were included. Reviews reporting on chemicals found in human biosamples of EC users were also included, since they reflect user exposure to chemical constituents present in ECs. EC devices and e-liquids of all types were considered.

### 2.3. Data Extraction

Two reviewers (NT, MK) developed a standardized data extraction tool. Extracted information from reviews included: citation, authors’ financial disclosures, review objectives, details of the literature search, designs of included studies, description of exposure (e.g., duration of exposure, EC device, e-liquid or aerosol), outcome variables, cell/tissue types, quality appraisal tool used, key findings, authors’ concluding views along with recommendations, and reported study limitations. Data from each review was extracted and documented by one reviewer (MK), and checked for completeness and accuracy by the second reviewer (NT).

### 2.4. Assessment of Quality and Risk of Bias

Included reviews were independently appraised for quality and bias by two reviewers (NT and MK) using the AMSTAR-2 checklist [12]. The description and the exact application of this tool in the framework of our umbrella review have been previously published [7].

## 3. Results

**Figure 1:**
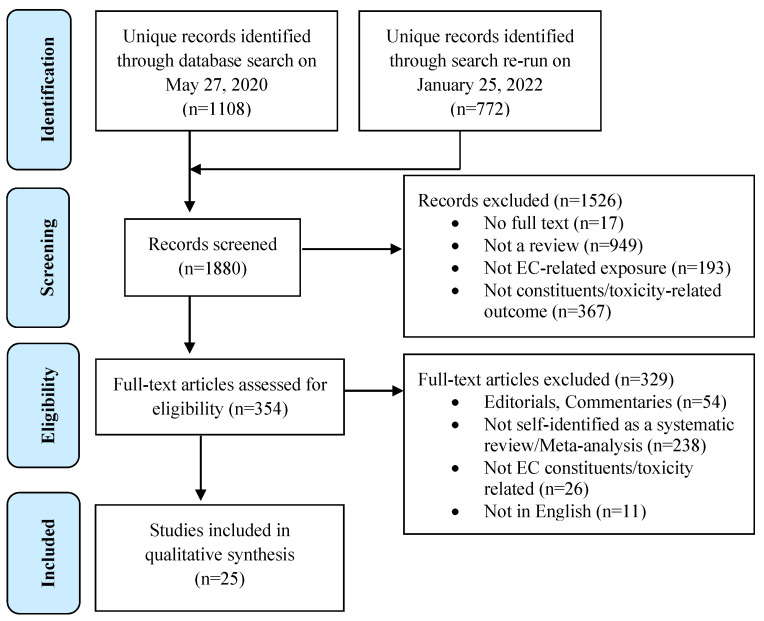
PRISMA flow diagram of the study selection process for the umbrella review.

We identified 1108 individual articles from the databases searched on 27 May 2020, and an additional 772 unique records were found through our re-run search conducted on 25 January 2022 (Figure 1). 

Full-text appraisal was conducted for 354 of these studies. The most common reason for excluding full texts was the unfulfilled criterion of being a systematic review or meta-analysis (*n* = 238). Twenty-five systematic reviews including one meta-analysis were deemed eligible for inclusion in our study. Chemical profiles of ECs were investigated in twenty [6,13,14,15,16,17,18,19,20,21,22,23,24,25,26,27,28,29,30,31] and the toxicity of ECs was covered in fourteen reviews [13,15,18,19,21,23,26,28,30,32,33,34,35,36]. Nine reviews investigated both outcomes [13,15,18,19,21,23,26,28,30]. Table 1 presents the characteristics of the included reviews. 

### 3.1. AMSTAR 2 Assessment of Quality and Risk of Bias

Table 2 shows the results from the AMSTAR-2 Assessment tool. Twenty-three reviews failed to report whether their review methods were established in a written protocol prior to conducting the review, as recommended by the PRISMA Guidelines [11]. Only five reviews applied an appropriate technique for systematically assessing the potential risk of bias within their included studies, and only four accounted for the risk of bias in individual studies when interpreting their results. Twenty-three reviews disclosed their sources of funding, while only eight reviews reported information on funding and any conflicts of interest for the included studies. Fourteen reviews did not apply a comprehensive literature strategy, and only one review received full credit for this domain (e.g., searching at least two databases, searching reference lists of included studies, trial/study registries). In nine reviews, the study selection was performed in duplicate and seven reported to have performed data extraction in duplicate. None of the reviews provided a list of excluded studies with justifications for exclusion of each potentially relevant study.

### 3.2. Chemical Profiles of E-Liquids and EC Aerosols

Twenty reviews investigated the chemical composition of e-liquids and EC aerosols [6,13,14,15,16,17,18,19,20,21,22,23,24,25,26,27,28,29,30,31]. Results were generally reported without a clear stratification between constituents found in e-liquid vs. EC aerosols. Below, we focus on the constituents that were most commonly reported across the reviews. 

#### 3.2.1. Metals/Metalloids

Twelve reviews examined chemical evaluations of metals and metalloids (i.e., semimetals) in e-liquids and EC aerosols [6,14,15,16,19,21,22,23,25,26,30,31]. Reviews consistently found evidence of these elements being present in ECs, although considerable heterogeneity was reported in the detected levels across the included studies [6,14,15,16,19,21,22,23,25,26,30,31]. Higher levels of metals were seen in EC aerosols after e-liquids came in contact with the heating coil and metal components of the EC device, while metal levels varied by brand and device characteristics, such as power and coil material [6,14,16,21,22,23,31]. Two reviews reported that the levels of metals/metalloids in ECs were significantly lower than in CC smoke, while one suggested that the levels in ECs may be higher, likely due to the metal components of EC devices [16,19,21]. Reviews of studies examining human biosamples confirmed similar or higher levels of metals and metalloids in EC users’ urine and serum compared to CC or cigar users [14,26].

#### 3.2.2. Carbonyls

Carbonyl levels were examined in 15 reviews [6,15,18,19,20,21,22,23,24,25,26,28,29,30,31]. Carbonyls such as formaldehyde and acetaldehyde were generally found at lower levels in EC aerosols and e-liquids compared to CCs, with levels substantially differing between and within EC samples depending on device type and the specific study [6,15,18,19,20,21,23,24,25,26]. In particular, aerosol emissions from fourth generation nicotine-salt-containing devices, such as Juul, were shown to contain lower levels of carbonyls when compared to other ECs and CCs [28]. Carbonyl concentrations were found to be positively correlated to higher power levels and temperatures of EC devices [6,20,23,24,25,30,31]. 

#### 3.2.3. Other Common Constituents

Volatile organic compounds (VOCs), tobacco-specific nitrosamines (TSNA), N’-Nitrosonornicotine (NNN), 4-(methylnitrosamino)-1-(3-pyridyl)-1-butanone (NNK), and polycyclic aromatic hydrocarbons (PAHs) were among other common chemical constituents measured in chemical profile analyses within 15 reviews [6,13,15,17,18,21,23,25,26,27,28,29,30,31,36]. Reviews found that, while present in many e-liquid and EC aerosol samples, the levels of these chemicals were often below the level of detection or significantly lower than levels found in CCs [6,13,15,17,18,21,23,25,26,28,31]. However, while levels were lower, it was commonly found across reviews that levels widely varied depending on study techniques and were inconsistent with EC labeling [6,15,17,18,21,23,25,26,31]. Notably, reviews emphasized the difficulty in identifying true exposure to carcinogenic compounds due to user-adjustable operating parameters of EC devices and unregulated e-liquids [29,30]. Similarly, reviews that included human experimental studies found the presence of NNN, NNK, and other carcinogens in the saliva, urine, and blood of EC users, though most often at lower levels than that of CC users [13,26,27]. When looking at flavored e-liquids, 173 chemical compounds were identified with ester and alkene as the most frequent chemical class, while aryl, alcohol, ketone, aldehyde, and lactone were among other common chemical classes identified [29]. Predictions of chemical transformations of these flavoring components upon aerosolization suggest their possible correlation to aerosol toxicants [29].

### 3.3. Toxicity of ECs

Fourteen reviews covered studies investigating the toxicity of ECs, mostly relying on evidence from studies exposing human cell cultures to EC aerosol and/or e-liquid [13,15,18,19,21,23,26,28,30,32,33,34,35,36]. Reviews consistently found that, upon exposure to EC aerosol and e-liquid, there was evidence of reduced cell viability, increased apoptosis, DNA damage, oxidative stress, impaired immune function, and an increase in inflammatory cytokines [13,15,18,19,21,23,26,28,30,32,33,34,35]. Generally, reviews with studies evaluating e-liquids reported more cytotoxic effects compared to EC aerosols. One review found evidence that while exposure to e-liquid and/or EC aerosol was associated with cytotoxicity, oxidative stress, reduced viability, delayed fibroblast migration, aberrant morphology, and genotoxicity, cigarette smoke exposure was found to be significantly more toxic to human cell cultures [34]. 

#### Flavoring Additives

Reviews that included evidence from studies testing the toxicity of flavored e-liquids reported cytotoxic effects of flavoring additives [13,15,16,17,18,19,21,26,30,31,32,33,35,36]. Among flavors, cinnamon [15,17,18,19,21,30,31,33,35] and menthol/mint [13,30,32,33] were singled out for their toxic properties (i.e., increased cell necrosis, altered cell morphology). One review in particular found sweet, fruity, and citrus flavored e-liquids to be more damaging to human tissues (e.g., initiate pathological processes, oxidative stress) compared to vanilla-flavored or non-flavored e-liquids [35]. Diacetyl and acetyl propionyl, artificial butter flavoring agents used in e-liquids, have been associated with an increased risk of developing bronchiolitis obliterans, a pulmonary disease previously detected among microwave popcorn industry workers [19,30,31,36]. 

### 3.4. Main Conclusions of Systematic Reviews

Due to the variable nature of product use among EC users and varying design parameters, most reviews that examined EC toxicity concluded that evidence is still too limited to draw definite conclusions about the potential human health effects of ECs, and that while ECs are not innocuous at the cellular level, they are likely much safer than CCs [6,13,15,17,18,19,20,21,22,23,24,25,26,30,31,32,33,34,36]. However, there was an agreement among several reviews that the evidence of negative impact on health warrants stronger regulation of ECs [14,21,28,35]. One review offered a more cautious interpretation of current evidence, determining that there is insufficient quality research to conclude that ECs are less harmful than CCs [32]. Similarly, another review specifically reviewing new generation EC devices, such as Juul, found that while e-liquids and aerosols from such devices may contain less harmful constituents than other types of e-cigarettes and cigarettes, there is no evidence that these levels are safe for young adults [28]. Two reviews concluded that ECs are a major concern for exposure to metals/metalloids, questioning whether they should even be marketed as a safer alternative to CCs [14,16]. While the evidence on the toxic effects of flavoring additives is limited to e-liquid testing, one review concluded that there is a possible correlation of flavor compounds in e-liquids to aerosol toxicants, calling for a conceptual framework that can further enhance this knowledge [29]. 

### 3.5. Main Limitations of Current Research

Authors of the included reviews identified several methodological limitations within their included studies. A major limitation mentioned in nearly half of the reviews was the lack of validated and standardized methods for collecting evidence on chemical profiles and toxicity evaluations that could explain variations in the estimated levels of toxicants [6,14,15,16,19,20,23,26,31,32,35,36]. Further, reviews emphasized that methods currently used to evaluate chemical compositions and cellular toxicity do not necessarily reflect actual exposure from realistic EC use behaviors in humans [6,18,19,21,22,25,29,31,35,36]. Thus, authors cautioned directly applying findings to in vivo situations. Evidence from human biosample studies included in some reviews were deemed weak by review authors due to small sample sizes, lack of control groups, missing definitions of smoking status, and short exposure periods [13,14,15,19,26,27]. Several reviews expressed concern that most research lacked information on devices used, and, those that did, often included evaluations from first-generation devices which may not reflect current consumer use [6,26,27].

### 3.6. Recommendations of Systematic Reviews

Future recommendations from authors of reviews examining chemical profiles of ECs included standardizing laboratory protocols for methods of extracting compounds from EC aerosols and e-liquids, and uniform units of detection [6,14,18,19,20,21,25,31]. One review suggested that future experimental research should address typical EC use behavior so that accurate puff intervals can be established [22]. Additionally, several reviews highlighted the need to emphasize the importance of conducting long-term in vivo studies, which is essential for understanding true human exposure [13,21,24,26,28,32,36]. One review emphasized the importance of focusing on both exclusive EC users and dual EC and CC users in future research in order to better distinguish health consequences of ECs [33]. Methods of validation and identification of appropriate control groups and relevant biomarkers were among other recommendations [13,19]. Continued testing of the toxicity of flavored EC aerosols and e-liquids was also a common recommendation [13,20,21,29], with two reviews explicitly recommending that diacetyl should not be used as a flavoring component in e-liquids [26,36]. In order to improve the safety profiles of ECs, one review suggested that regulatory agencies play a central role in guiding the acceptable levels of exposure to the various constituents [31]. With new products constantly emerging, reviews generally recommended focusing future research on newer generations and models.

## 4. Discussion

This umbrella review summarized evidence of the chemical constituents and in vitro toxicity of ECs from existing systematic reviews while highlighting the limitations of current research. Wide variations were reported in the chemical compositions of ECs across studies included in reviews, likely due to a lack of standardized protocols for collecting evidence on chemical profiles and the heterogeneity of devices and e-liquids evaluated. Reviews generally found that metals/metalloids were more abundant in EC aerosols than in e-liquids due to contact with a heating coil and metal components of the device. Carbonyls and other constituents (e.g., tobacco-specific nitrosamines, polycyclic aromatic hydrocarbons) were consistently found at lower levels in ECs than in CCs. Reviews found evidence of in vitro toxicity of ECs, although to a lesser extent than CCs, with more cytotoxic effects reported for e-liquids compared to EC aerosols. Notably, evidence from human cell culture studies showing significantly higher cytotoxicity of cigarette smoke compared to EC aerosol has been well documented in the past [37,38]. Among flavoring additives in e-liquids, cinnamon and menthol/mint were found to be particularly cytotoxic. However, considerations of dose, mode of administration, and lack of human in vivo data leave unanswered the clinical importance of these findings.

The need for standardized and validated protocols for chemical profile and toxicity studies was a predominant theme in the limitations and discussions across nearly all included reviews. While approaches for testing combustible tobacco products are relatively well established, approaches for ECs are less developed [9]. Because of the large and constantly evolving varieties of ECs, developing standard protocols is challenging, especially for toxicity assessments [39,40]. Recently, tools and frameworks have been proposed to standardize puffing patterns and toxicology assessments [40,41]. In particular, rigorous analytical approaches for accurate assessment of the concentrations of toxic metals in EC aerosols have been proposed and validated as part of good inorganic analytical practice to help eliminate possible sources of leachable metal background in the sample collection materials [42,43]. The use of such validated methods would enable future research to better understand the relative chemical profiles and toxicity of ECs and improve the validity and comparability of research findings.

A lack of transparency in the methods of the included studies was another major concern across the reviews. Inadequate detail regarding the EC device types limited the ability to compare results across studies. Importantly, reviews found that the identified constituent levels often deviated from those reported on labels. More research is needed to identify if these deviations are due to inaccurate measurement methods or poor labeling standards. Future reviews should focus on the degree to which ECs are less toxic than CCs, with clear interpretation as to how these findings can be translated into regulatory and public health guidelines. Furthermore, reviews should compare the different classes of EC products in terms of their toxicity, the determinants of toxicity, and explore steps that could be taken to reduce such toxicity. For this purpose, future reviews may also consider unpublished, but otherwise publicly available, data on toxicity and chemical constituents as submitted by manufacturers to FDA in their pre-market and modified-risk applications.

The abundance of available flavored e-liquids on the market and the lack of mandatory labeling of flavoring agents further extend the question of safety of ECs. While some country- and market-specific EC flavor classifications have been proposed, a universal classification system of EC flavoring compounds is needed [44,45]. With most available studies on the toxicity of flavoring additives being restricted to e-liquids, future research should focus on how these additives contribute to toxicant emissions in EC aerosols. Additionally, more in vivo studies are needed to better understand the toxic health effects of EC use on humans.

While reviews identified common limitations of the current empirical research, our study found important methodological weaknesses of these systematic reviews. The assessment of methodological quality and risk of bias using the AMSTAR-2 checklist found many incomplete domains, indicating major methodological flaws and a potentially high risk of bias in the included systematic reviews. As such, over 90% of the included reviews failed to report whether their review methods were established in a written protocol prior to conducting the review, despite recommendations by Cochrane Collaboration, the National Academy of Medicine, and PRISMA guidelines [11,46,47]. This finding is even more staggering compared to a study that found that only one-third of systematic reviews published in MEDLINE in 2014 reported working from a previously developed protocol [48]. Detailed review protocols, developed a priori, are crucial to ensure that the chosen research designs/methods are appropriate, and that the findings of the reviews are adequately reported. Registering protocols in a database for systematic reviews enables researchers to better communicate their research aims and methods, prevents the unintentional reproduction of reviews addressing the same topic, and allows researchers to reproduce and validate findings or properly update the review with new evidence. Further, 80% of the systematic reviews failed to use an appropriate technique to assess the risk of bias of the included studies, and more than half of the reviews did not apply a comprehensive literature search strategy when conducting their review. Such methodological gaps may have resulted in reviews including studies with design flaws and prevented reviews from adequately capturing available evidence. Future systematic reviews should therefore strictly adhere to the established guidelines to minimize their risk of bias and avoid magnifying “waste” from primary studies.

To our knowledge, this is the first umbrella review to have focused on chemical profiles and toxicity of ECs. While this umbrella review provides a comprehensive and reproducible synthesis of the available literature, it has several limitations. Consistent with the first item on the PRISMA 2021 Checklist [11], our review identified systematic reviews as those that explicitly stated that they were “systematic reviews” in their title, abstract, methods, or keyword. This approach was selected as the most straightforward and transparent way to detect a systematic review. However, it may have led to the exclusion of well-designed literature reviews that did not self-identify as systematic, while at the same time allowing for the inclusion of reviews with methodological shortcomings, as indicated by the AMSTAR-2 checklist. We chose the AMSTAR-2 quality appraisal tool to be most suitable for our umbrella review among other existing tools [49]. However, in light of the heterogeneity in the methods and outcomes of the included reviews and due to the previously observed abundance of critically low ratings produced by the tool [50], we chose not to assign the overall quality ratings for systematic reviews. Instead, our approach was to narratively describe the areas of potential bias exhibited in our included reviews. Further, our literature search did not impose a restriction on publication date. Consequently, our analysis included seven reviews with data collection up to 2015, before new-generation ECs devices, such as Juul of Puff Bar, entered and subsequently dominated the U.S. market. Thus, their findings may be of limited relevance in the rapidly growing and diversifying EC market. Stratification of our findings by EC device generation or e-liquid type was not possible due to underreporting of their characteristics in most reviews. Further, given the abundance of chemical constituents present in e-liquids and aerosols, our reviews only focused on the constituents that were most commonly reported in the included systematic reviews. Due to the limited comparability of reviews, summarizing the evidence in quantitative terms was beyond the scope of this umbrella review. Finally, since we did not specifically seek to identify the overlapping original studies included in the reviews due to the large volume, some studies may be potentially overrepresented in our umbrella review.

## 5. Conclusions

This umbrella review examined evidence of the chemical composition and in vitro toxicity of ECs and highlighted methodological limitations of current research. It generally found evidence of toxicity of ECs, although to a lesser extent than CCs. It also identified the most common chemical constituents in ECs, but there was wide variation in the exact composition of EC aerosols and e-liquids within and across the reviews. This variation is mainly attributed to the differences in EC devices and e-liquids tested, and the lack of standardized protocols for methods of analyzing the chemical profiles of ECs. With EC products constantly evolving and their potential health effects taking years to assess, chemical and in vitro studies will continue to be essential for policymakers and regulatory agencies as they grapple with the issues of evaluating and regulating current and future EC products. Our study highlights the need for future systematic reviews on the composition and toxicity of ECs with better adherence to established reporting guidelines to adequately inform tobacco regulatory actions. The clinical significance of EC toxicity and the difference in toxicity between the types of e-cigarettes and in relation to combustible cigarettes should be the focus of future research.

## Figures and Tables

**Table 1:** Characteristics of Included Systematic Reviews.

Characteristics		Citations
**Funding Source**	***n* = 25**	
Federal	11	[6,14,18,19,22,23,27,28,29,33,35]
Non-Tobacco Affiliated Organization	4	[19,28,31,36]
Tobacco Affiliated Organization	3	[18,20,25]
No Specific Grant from Any Funding Agency	5	[13,15,21,30,34]
Not Stated	5	[16,17,24,26,32]
**Number of Studies Included**	***n* = 25**	
Not stated	1	[36]
<10	1	[22]
10–50	15	[6,13,14,16,17,20,23,24,27,28,29,30,32,33,34]
51–100	7	[15,18,19,21,25,31,35]
100+	1	[26]
**Number of Databases Searched**	***n* = 25**	
1	11	[17,18,20,21,22,24,25,26,29,30,35]
2	2	[23,33]
3	5	[13,14,15,16,36]
4+	7	[6,19,27,28,31,32,34]
**Sample Type**		
**Chemical Profiles** E-liquid/EC Aerosol	***n* = 20**	
	18	[6,14,15,16,17,18,19,20,21,22,23,24,25,26,28,29,30,31]
Biosample	8	[13,14,15,16,18,19,26,27]
**Toxicity**	***n* = 14**	
Human Cells	13	[13,15,18,19,21,23,26,28,32,33,34,35,36]
Animal Cells	6	[15,19,21,26,32,35]
Not stated	1	[30]

A detailed summary of the included reviews can be found in Supplementary Tables S2 and S3.

**Table 2:** AMSTAR-2 Quality Assessment Scores; Chemical Profiles and Toxicity Outcomes.

	Research Question Included PICO	Review Methods Established Prior to Review *	Explanation of Study Design Selection	Comprehensive Literature Search Strategy *	Study Selection Performed in Duplicate	Data extraction Performed in Duplicate	List of Excluded Studies with Justification *	Describe Studies in Adequate Detail	Satisfactory Technique for Assessing Rob RCT/NRSI *		Report Sources of Funding	Use appropriate Methods for Statistical Combination of Results (for meta-Analysis) RCT/NRSI *	Assess potential Impact of RoB in Individual Studies on the Results (for Meta-Analysis)	Account for RoB in Individual Studies When Interpreting Results *	Explanation for and Discussion of Heterogeneity in Results	Investigate Publication Bias and Discuss Impact (for Quantitative Synthesis) *	Reported Any Conflict of Interest
Farsalinos and Polosa, 2014 [21]												NA	NA			NA	
Burstyn, 2014 [25]									NA			NA	NA			NA	
Cheng, 2014 [6]												NA	NA			NA	
Harrell et al., 2014 [23]												NA	NA			NA	
Pisinger and Dossing, 2014 [15]									NA			NA	NA			NA	
Ioakeimidis et al., 2016 [24]												NA	NA			NA	
Glasser et al., 2017 [18]												NA	NA			NA	
Zulkifli et al., 2018 [22]									NA			NA	NA			NA	
Farsalinos and Gillman, 2018 [20]												NA	NA			NA	
Kaur et al., 2018 [19]												NA	NA			NA	
Flach et al., 2019 [32]									NA			NA	NA			NA	
Gaur and Agnihotri, 2019 [16]												NA	NA			NA	
Armendáriz-Castillo et al., 2019 [17]												NA	NA			NA	
Bjurlin et al., 2020 [27]												NA	NA			NA	
Bozier et al., 2020 [26]												NA	NA			NA	
Lee et al., 2020 [28]												NA	NA			NA	
§ Salam et al., 2020 [29]	NA								NA								
Sharma and Verma, 2020 [30]												NA	NA			NA	
Ward et al., 2020 [31]	NA								NA			NA	NA			NA	
Zhao et al., 2020 [14]									NA			NA	NA			NA	
Yang et al., 2020 [13]												NA	NA			NA	
Bravo-Gutierrez et al., 2021 [35]												NA	NA			NA	
White et al., 2021 [36]												NA	NA			NA	
Wills et al., 2021 [33]									NA			NA	NA			NA	
Wilson et al., 2022 [34]												NA	NA			NA	

* Critical domains. § Meta-analysis. Green = yes; red = no; orange = partial yes. PICO = Population or participants, and conditions of interest; Interventions or exposures; Comparisons or control groups; Outcomes of interest; RCT = randomized controlled trials; NRSI = non-randomized studies of interventions; RoB = risk of bias; NA = not applicable. One review cautioned that several of their included studies heated e-liquids at a power level beyond the capability of most modern EC systems, potentially attributing to high levels of carbonyls [25]. Potentially higher carbonyl levels were also reported in e-liquid or EC aerosol samples that used vegetable glycerin solvent compared to propylene glycol solvent in one review [20].

## Data Availability

Not applicable.

## Supplementary Materials

The following supporting information can be downloaded at: https://www.mdpi.com/article/10.3390/ijerph20031908/s1, Table S1: The search strategy for the PubMed database; Table S2: Characteristics of Sys-tematic Reviews Including Chemical Profiles of ECs (*n* = 20); Table S3: Characteristics of Systematic Reviews Including Toxicity of ECs (*n* = 14).
