# Supplementary material for: Chemical Profiles and Toxicity of Electronic Cigarettes: An Umbrella Review and Methodological Considerations
Source: Int J Environ Res Public Health. 2023 Jan 20;20(3):1908. doi: 10.3390/ijerph20031908 (PMC9914618; doi:10.3390/ijerph20031908)
Supplement: Supplementary file 1 [file ijerph-20-01908-s001.zip › ijerph-2038646-supplementary.pdf]

Chemical Profiles and Toxicity of Electronic Cigarettes: An Umbrella Review and Methodological Considerations

Nargiz Travis, MSPH[1], Marie Knoll, MSPH[1], Steven Cook, PhD[2], Hayoung Oh, MPH[1], Christopher J. Cadham, MPH[3], Luz María Sánchez-Romero, PhD[1], David T. Levy, PhD[1]

[1]Lombardi Comprehensive Cancer Center, Georgetown Medical University, Washington, DC

[2]Department of Epidemiology, School of Public Health, University of Michigan, Ann Arbor, MI

[3]Department of Health Management and Policy, School of Public Health, University of Michigan, Ann Arbor, MI

Supplementary Table S1. Search strategy for the PubMed Database.

**PubMed**		
**Date Searched**	**Search String**	**Results**
Through May 27, 2020	("electronic cigarette" OR "e-cigarette" OR "electronic nicotine delivery system" OR "personal vaporiser" OR "personal vaporizer" OR ("e-liquid" AND "nicotine content")) AND ("systematic review" OR "meta-analysis" OR "review")	418
Between May 27, 2021 through January 25, 2022	("electronic cigarette" OR "e-cigarette" OR "electronic nicotine delivery system" OR "personal vaporiser" OR "personal vaporizer" OR ("e-liquid" AND "nicotine content")) AND ("systematic review" OR "meta-analysis" OR "review")	299

Supplementary Table S2. Characteristics of Systematic Reviews Including Chemical Profiles of ECs (n=20).

Author/Year	Funding Source/ Conflict of Interest	Sample type	EC device type	E-liquid type	Number of studies included	Databases searched	Date range of search	Quality Appraisal Tool used
Armendáriz-Castillo et al., 2019	Not stated/ The authors declare that they have no conflict of interest	**Chemica**l: EC aerosols and liquids	Not specified	Not specified	10	PubMed	not specified	None reported
Bjurlin et al., 2020	Supported by the National Center for Advancing Translational Sciences (NCATS), National Institutes of Health, through Grant Award Number UL1TR002489 (MAB), and a New York State ECRIP's award. Authors declare no conflicts of interest.	**Biosample**: urine	Most included studies did not report or differentiate between older and newer devices	Not specified	22	PubMed, Embase, Web of Science, and Cochrane Central Register of Controlled Trials (CENTRAL)	Through January 2019	None reported
Bozier et al., 2020	None declared	**Chemical**: EC liquids and aerosols **Biosample:** urine and blood	Not specified	Included flavor and nicotine-containing	225 in total for all outcomes (no further breakdown)	PubMed	February 2017 through May 2019	None reported
Burstyn, 2014	Funding for this work was provided by The Consumer Advocates for Smokefree Alternatives Association (CASAA) Research Fund. The author, not the funder, had full control of the content.	**Chemical:** EC liquids and aerosols from smoking machines or volunteer vapers	Not specified	Not specified	59	PubMed	through July 2013	None reported
Cheng, 2014	Not stated/No competing interests	**Chemical:** EC refill solutions, aerosols, and environmental emission of ECs	Not specified	Included nicotine-containing refill solutions, cartiges	29	Web of Knowledge, PubMed, SciFinder, Embase and EBSCOhost	January 2007 - September 2013	None reported

Farsalinos & Polosa, 2014	This research received no specific grant from any funding agency in the public, commercial, or not for-profit sectors/ Riccardo Polosa is a Professor of Medicine and is supported by the University of Catania, Italy. He has received lecture fees and research funding from GlaxoSmithKline and Pfizer, manufacturers of stop smoking medications. He has also served as a consultant for Pfizer and Arbi Group Srl (Milano, Italy), the distributor of Categoria™ ECs. His research on ECs is currently supported by LIAF (Lega Italiana AntiFumo). Konstantinos Farsalinos is a researcher at Onassis Cardiac Surgery Center. He has never been funded by the pharmaceutical or the tobacco industry. For some of his studies, the institution has received financial compensation from electronic cigarette companies for the studies' cost. His salary is currently being paid by a scholarship grant from the Hellenic Society of Cardiology	**Chemical:** EC liquid and aerosols	Mainly first-generation	Not specified	82	PubMed	None reported	None reported

Farsalinos & Gillman, 2018	In the past 3 years, KF has published 2 studies funded by the non-profit association AEMSA and 1 study funded by the non-profit association Tennessee Smoke-Free Association. Enthalpy Analytical is a for-profit CRO involved in analytical testing of tobacco and EC products.	**Chemical:** EC liquids and aerosols	First, second and newer generations	Not specified	32	PubMed	No date restriction	None reported
Gaur & Agnihortri, 2019	Not stated/ The authors declare that they have no conflict of interest	**Chemical:** EC aerosols and liquids **Biosample:** urinary metabolite profile	Not specified	Not specified	12	Medline (PubMed), Scopus, and Web of Science.	None reported	None reported
Glasser et al., 2017	The Schroeder Institute at Truth Initiative, the Robert Wood Johnson Foundation (Grant ID: 72208 and 72390), and a NIH K01 Career Development Award in Tobacco Control Regulatory Research (Principal Investigator, Pearson; 1K01DA037950-01)/All authors are employed by Truth Initiative	**Chemical**: Liquids and mainstream and exhaled EC aerosol **Biosample:** human EC and CC blood samples	Not specified	Not specified	75	PubMed	through May 31, 2016	None reported
Harrell et al., 2014	National Cancer Institute Behavioral Oncology Training Grant (R25CA090314) at Moffitt Cancer Center in Tampa, FL, awarded to Paul Jacobsen, and by grants R01CA134347 and R01CA154596, awarded to Thomas Brandon and Vani Simmons, respectively / Thomas Brandon receives research support from Pfizer, Inc.	**Chemical:** EC liquid and aerosol	EC brands were documented, where possible.	Nicotine-free and nicotine-containing.	40	PubMed, Web of Science	through November 20, 2013	None reported
Ioakeimidis et al., 2016	Not stated	**Chemical:** EC liquid and aerosol	First and new-generation	Not specified	20	PubMed	through June 2015	None reported

Kaur et al., 2018	Supported by a Young Clinical Scientist Award from the Flight Attendant Medical Research Institute (FAMRI; 123253_YCSA_Faculty); National Institutes of Health/ R15 (7 R15 ES023151 02); a Southern University Foundation Grant (FY2017-017); and a Louisiana Biomedical Research Network Startup Grant (2P20GM103424-14 Subaward No. 100011) to S. Batra / No competing interests	**Chemical:** EC aerosol and liquid **Biosample**: plasma nicotine levels, serum cotinine levels	Not systematically reported	Included nicotine and flavor containing	56	PubMed, BioMed Central, US FDA, Centers for Disease Control and Prevention, the JAMA network and the Agency for Toxic Substances and Disease Registry	1980-2017	None reported
Lee et al., 2020	This research was made possible by a gift from Roslyn and Lisle Payne. This research was also supported by grants R25CA057711 and 2T32CA057711-26 from the National Cancer Institute of the National Institutes of Health/ Dr. Rees reported grants from the National Cancer Institute and personal fees from expert testimony in tobacco litigation outside the submitted work.	**Chemical**: pod-based EC liquids and aerosols	Pod-based ECs (i.e., JUUL)	Included nicotine-containing	35	PubMed, Web of Science, Embase, and EBSCO HOST	between June 2015 to March 15, 2019	None reported
Pisinger & Dossing, 2014	This research received no specific grant from any funding agency in the public, commercial or not-for-profit sectors/ The authors have no conflict of interest	**Chemical:** EC liquid or aerosol **Biosample**: saliva	Not specified	Included nicotine and flavor-containing	76	PubMed, EMBASE and CINAHL	through 14 Aug 2014	None reported

Salam et al., 2020	Supported by grant number U54DA036105 from the National Institute on Drug Abuse of the National Institutes of Health and the Center for Tobacco Products of the US Food and Drug Administration/ Drs. Eissenberg and Shihadeh are paid consultants in litigation against the tobacco industry and the electronic cigarette industry. They are named on a patent for a device that measures the puffing behavior of electronic cigarette users. In addition, Dr. Eissenberg is named on another patent for a smartphone app that determines electronic cigarette device and liquid characteristics.	**Chemical:** EC liquids	Not specified	Flavor-containing	11	PubMed	through November, 2019	None reported
Sharma & Verma, 2020	None/None	**Chemical**: EC liquids and aerosols	Not specified	Included nicotine and flavor-containing	36	PubMed	published in the last 5 years-published 2021	None reported
Ward et al., 2020	Funded by HH Sheikh Hamed Bin Zayed Al Nahyan/ JOE declares that he has received consulting fees and is a shareholder in Nesmah / The authors have no other conflicts of interest to declare	**Chemical**: EC aerosols	Variations in device characteristics, such as power, voltage, resistance, temperature, coil material, brand	Included flavor-containing	92	Ovid MEDLINE, Epub Ahead of Print, In-Process & Other Non-Indexed Citations, Daily and Versions electronic databases	1946- May 07, 2020.	None reported

Yang et al., 2020	The authors declare that there are no conflicts of interest	**Biosample:** collected saliva and other unspecified biosamples	EC brands were documented, where possible.	Included nicotine and flavor-containing	20	PubMed/, Web of Science, Embase	through December 2019	Effective Public Health Practice Project (EPHPP) Quality Assessment Tool for interventional studies. A modified version was used for noninterventional studies.
Zhao et al., 2020	This study was supported by NIEHS/FDA grants R21ES029777 and R01ES030025, NIEHS grant P30ES009089, and a Johns Hopkins University Technology Transfer Seed Award. D.Z. was supported by the China Scholarship Council (201706190116). A.A. was supported by a grant from the MD Cigarette Restitution Fund (grant PHPA-G2034)/The authors declare they have no actual or potential competing financial interests	**Chemical**: EC liquids and aerosols **Biosample**: human biosamples from EC users (urine, saliva, serum, and blood)	Cig-a-likes, tanks.	Bottle, cartridge, open wick tank e-liquids, including flavor and nicotine-containing	24	Pubmed/TOXLINE, Embrase, Web of Science	January 2008-19 July 2018	Adapted QualSyst Tool for systematic reviews of quantitative studies
Zulkifli et al., 2018	This review paper was made possible because of the financial support from the Fundamental Research Grant Scheme (FRGS) Ministry of Education Malaysia, under the Vote 5524532 and Ministry of Higher Education Malaysia (MyBrain) / None	**Chemical:** Chemicals and heavy metals in ECs	EC brands were documented, where possible.	Not specified	4	PubMed	2000- August 2015	None reported

Supplementary Table S3. Characteristics of Systematic Reviews Including Toxicity of ECs (n=14).

Author/Year	Funding Source/ COI	Sample type	EC device type	E-liquid type	Number of studies included	Databases searched	Date range of search	Quality Appraisal Tool used
Bozier et al., 2020	Not stated/None	H**uman:** lung cells, gingival crevicular fluid, sulcar fluid, and saliva of EC users **Animal**: mouse cytokine data	Not specified	Included flavor and nicotine-containing	225 in total for all outcomes (no further breakdown)	PubMed	February 2017 -May 2019	None reported
Bravo-Gutierrez et al, 2021	This work was supported by the allocated budget to research (R. F-V-HLA Laboratory) from the Instituto Nacional de Enfermedades Respiratorias Ismael Cosío Villegas (INER).	**Human:** immortal cell lines from different parts of the lung, human bronchial-epithelial (HBE) cells obtained from healthy volunteers **Animal:** lungs of mice and rats	Not specified	Included flavor and nicotine-containing	79	PubMed	January 2013-August 2020	None reported
Farsalinos & Polosa, 2014	No funding/ Riccardo Polosa is a Professor of Medicine and is supported by the University of Catania, Italy. He has received lecture fees and research funding from GlaxoSmithKline and Pfizer, manufacturers to stop-smoking medications. He has also served as a consultant for Pfizer and Arbi	**Human:** embryonic stem cells, pulmonary fibroblasts, fibroblast cells, cardiomyoblasts. **Animal:** neural stem cells.	Mainly 1st generation	Not spesified	82	PubMed	Not specified	None reported

	Group Srl (Milano, Italy), the distributor of Categoria™ ECs. His research on ECs is currently supported by LIAF (Lega Italiana AntiFumo). Konstantinos Farsalinos is a researcher at Onassis Cardiac Surgery Center. He has never been funded by the pharmaceutical or the tobacco industry. For some of his studies, the institution has received financial compensation from electronic cigarette companies for the studies' cost. His salary is currently being paid by a scholarship a grant from the Hellenic Society of Cardiology							
Flach et al., 2019	Not stated/None	**Human:** cells derived purely from the oral cavity, whereas one study worked with oropharyngeal cells and cells originated from a primary laryngeal tumor in addition to oral cells. **Animal:** rat cells (vocal cords).	Brands were documents, where possible	Included nicotine and flavor-containing	18	PubMed/MEDLINE, Cochrane, CINAHL Plus, Trip Medical Database and Web of Science	Through September, 2018	None reported

Glasser et al., 2017	The Schroeder Institute at Truth Initiative, the Robert Wood Johnson Foundation (Grant ID: 72208 and 72390), and a NIH K01 Career Development Award in Tobacco Control Regulatory Research (Principal Investigator, Pearson; 1K01DA037950-01)./All authors are employed by Truth Initiative	**Human:** cell viability, inhibitory concentration 50, human pulmonary fibroblast survival rate, pro-inflammatory mediators, adverse effect level, IL-6 protein, lactate dehydrogenase, Hrv rna and human splunc 1mrna, pro-inflammatory neutrophils, antimicrobial activity, adenylate kinase (no specification on type of cell)	Not specified	Not specified	116	PubMed	Through May 31, 2016	None reported
Harrell et al., 2014	National Cancer Institute Behavioral Oncology Training Grant (R25CA090314) at Moffitt Cancer Center in Tampa, FL, awarded to Paul Jacobsen, and by grants, R01CA134347 and R01CA154596, awarded to Thomas Brandon and Vani Simmons, respectively / Thomas Brandon receives research support from Pfizer, Inc.	**Human:** bronchial epithelial cells	EC brands were documented, where possible.	Nicotine-free and nicotine-containing.	4	PubMed, Web of Science	Through November 20, 2013	None reported

Kaur et al., 2018	Supported by a Young Clinical Scientist Award from the Flight Attendant Medical Research Institute (FAMRI; 123253_YCSA_Faculty); National Institutes of Health/ R15 (7 R15 ES023151 02); a Southern University Foundation Grant (FY2017-017); and a Louisiana Biomedical Research Network Startup Grant (2P20GM103424-14 Subaward No. 100011) to S. Batra./ None	**Human:** alveolar epithelial cells, keratinocytes, human umbilical cord vein endothelial cells, human primary airway epithelial cells from nonsmokers, human bronchial epithelial cells, human embryonic stem cells. **Animal:** mouse neural stem cells, mouse tracheal epithelial cells, lungs/	Not systematically reported	Included nicotine and flavor containing	56	PubMed, BioMed Central, US FDA, Centers for Disease Control and Prevention, the JAMA network and the Agency for Toxic Substances and Disease Registry	1980-2017	None reported
Lee et al., 2020	This research was made possible by a gift from Roslyn and Lisle Payne. This research was also supported by grants R25CA057711 and 2T32CA057711-26 from the National Cancer Institute of the National Institutes of Health/ Dr Rees reported grants from the National Cancer Institute and personal fees from expert testimony in tobacco litigation outside the submitted work.	**Human** cell (not specified)	Pod-based ECs (i.e., JUUL)	Included nicotine-containing	35	PubMed, Web of Science, Embase, and EBSCO HOST	June 2015 - March 15, 2019	None reported

Pisinger & Dossing, 2014	This research received no specific grant from any funding agency in the public, commercial or not-for-profit sectors/ none	**Human:** embryonic and stem cells, human pulmonary fibroblasts **Animal**: mouse neural cells, cultured murine fibroblasts	Not specified	Included nicotine and flavor-containing	4	PubMed, EMBASE and CINAHL	Through August 2015	None reported
Sharma & Verma, 2020	none/none	In vitro cells (not specified)	Not specified	Included nicotine and flavor-containing	36	PubMed	2016- 2021	None reported
White et al., 2021	ECU grant #111101/ none	**Human:** liver cells and other unspecified cell types	Not specified	Flavor-containing	Not stated	PubMed, Scopus and Google Scholar	Not stated	None reported
Wills et al., 2021	National Cancer Institute (grant: P30 CA071789). Funding information for this article has been deposited with the Crossref Funder Registry/ Will reports this as a potential COI	**Human:** unspecified types of cells	EC brands were documented, where possible.	Included flavor-containing	37	PsycINFO and PubMed	Through March 2020	None reported
Wilson et al., 2022	Not stated/None	**Human:** head, neck, and oral cells	Not specified	Included flavor and nicotine-containing	18	Medline, Dentistry and Oral Sciences, CINAHL, CAPLUS, Web of Science, and gray literature	Through December 2020	The study risk of bias table was adapted from an article on the National Center for Biotechnology Information web's site (NCBI)

Yang et al., 2020	The authors declare that there are no conflicts of interest	**Human:** cultured oral keratinocytes, human epithelial normal bronchial cells, human premalignant dysplastic oral mucosal keratinocyte cells, human oral squamous cell carcinoma cell, gingival epithelium cells, pulmonary epithelium cells, organotypic human buccal epithelial and small airway epithelial cells, normal human oral keratinocytes, human gingival fibroblasts, human periodontal ligament fibroblast, human gingival epithelium progenitors, human gingival tissue, mucosal tissue culture from fresh tissue samples of healthy oropharyngeal mucosa, head and neck squamous cell carcinoma	EC brands were documented, where possible	Included nicotine and flavor-containing	20	PubMed/Web of Science, Embase	Through December 2019	Effective Public Health Practice Project (EPHPP) Quality Assessment Tool for interventional studies. A modified version was used for noninterventional studies.
